# Pleiotropic Loci Associated With Foot Disorders and Common Periparturient Diseases in Holstein Cattle

**DOI:** 10.3389/fgene.2021.742934

**Published:** 2021-12-06

**Authors:** Ellen Lai, Alexa L. Danner, Thomas R. Famula, Anita M. Oberbauer

**Affiliations:** Animal Science Department, University of California, Davis, Davis, CA, United States

**Keywords:** pleiotropy, multivariate, genome-wide association study, dairy cattle, lameness, disease, genetic correlation

## Abstract

Lameness is an animal welfare issue that incurs substantial financial and environmental costs. This condition is commonly caused by digital dermatitis (DD), sole ulcers (SU), and white line disease (WLD). Susceptibility to these three foot disorders is due in part to genetics, indicating that genomic selection against these foot lesions can be used to reduce lameness prevalence. It is unclear whether selection against foot lesions will lead to increased susceptibility to other common diseases such as mastitis and metritis. Thus, the aim of this study was to determine the genetic correlation between causes of lameness and other common health disorders to identify loci contributing to the correlation. Genetic correlation estimates between SU and DD and between SU and WLD were significantly different from zero (*p* < 0.05), whereas estimates between DD and mastitis, DD and milk fever, and SU and metritis were suggestive (*p* < 0.1). All five of these genetic correlation estimates were positive. Two-trait genome-wide association studies (GWAS) for each of these five pairs of traits revealed common regions of association on BTA1 and BTA8 for pairs that included DD or SU as one of the traits, respectively. Other regions of association were unique to the pair of traits and not observed in GWAS for other pairs of traits. The positive genetic correlation estimates between foot disorders and other health disorders imply that selection against foot disorders may also decrease susceptibility to other health disorders. Linkage disequilibrium blocks defined around significant and suggestive SNPs from the two-trait GWAS included genes and QTL that were functionally relevant, supporting that these regions included pleiotropic loci.

## Introduction

Abnormal gait or posture in a cow are considered indicators of lameness and signifies pain and discomfort. Lameness is the second most prevalent disease after mastitis and the third most common reason for culling after mastitis and infertility (USDA, 2018). Lameness is commonly caused by foot lesions classified as infectious [e.g., digital dermatitis (DD), heel horn erosion, and foot rot] or noninfectious lesions [e.g., sole hemorrhage, sole ulcer (SU), white line disease (WLD), and laminitis]. Lameness not only raises welfare concerns, but also has economic and environmental consequences. Financial costs associated with lameness include direct costs for treatment and increased labor and indirect costs from reduced milk production and fertility; together, these costs range from $64 per case of DD to $178 per case of SU (Cha et al., 2010; Dolecheck and Bewley, 2018; Dolecheck et al., 2019). Reduced fertility, premature culling, and reduced milk production associated with lameness reduces the efficiency of resource use, as resources used for the cow are invested over a less productive and shorter lifetime, inflating the environmental costs per unit of milk by 14 (1.5%) kg CO_2_ equivalents per ton of fat-and-protein-corrected milk, on average (of DD, SU, and WLD combined) (Mostert et al., 2018).

Prevention of lameness is achieved through routine claw trimming, foot baths (for prevention of infectious lesions), maintaining floor hygiene, and nutrition. Despite these prevention efforts, lameness remains highly prevalent in the United States, affecting 16.8% of cows and 3.2% of bred heifers (USDA, 2018). These non-genetic methods of prevention can be aided by genetic selection, as implied by the low to moderate estimates of heritability for foot lesions, ranging from 0.01 to 0.4 for DD, 0.01 to 0.3 for SU, and 0.017 to 0.26 for WLD (Van der Waaij et al., 2005; Onyiro et al., 2008; van der Linde et al., 2010; Häggman and Juga, 2013; Oberbauer et al., 2013; van der Spek et al., 2013, 2015; Malchiodi et al., 2015; Biemans et al., 2018). Genetic selection uses prior knowledge about the contribution of certain genetic markers to traits of interest and creating a selection index reflecting a weighted average of multiple traits that is used to rank animals. Selective breeding programs utilize both genetic correlation among traits that are included in the selection index and specific susceptibility loci associated with the traits. Accordingly, selection against foot disorders would likely account for correlated lesion traits because some foot disorders are genetically correlated with each other, particularly within the infectious (strongest between DD and heel erosion) and noninfectious (strongest among sole hemorrhage, SU, and WLD) groupings of lesions (Koenig et al., 2005; Van der Waaij et al., 2005; van der Linde et al., 2010; Buch et al., 2011; Gernand et al., 2012; Häggman and Juga, 2013; van der Spek et al., 2013; Pérez-Cabal and Charfeddine, 2015; Malchiodi et al., 2017).

Additionally, certain foot lesions are genetically correlated with mastitis or indicator traits of mastitis. For example, the genetic correlations between clinical mastitis and sole hemorrhage or SU were estimated at 0.35 and 0.32, respectively, in Swedish Red cows (Buch et al., 2011). For Holstein cows, the genetic correlation between somatic cell score and individual foot lesions or lameness in general ranged from 0.15 to 0.24 (Koenig et al., 2005) and 0.23 (Gernand et al., 2012), respectively, although other studies failed to identify significant genetic correlations between DD or interdigital hyperplasia and clinical mastitis (Buch et al., 2011; Gernand et al., 2013). Nevertheless, the genetic correlation among foot disorders and between individual foot disorders and mastitis traits implies that common loci may coordinately influence these traits (Koenig et al., 2005; Buch et al., 2011). Such pleotropic loci have not been identified as of yet. The values for genetic correlation between foot and other health disorders that have been reported were estimated using pedigree information. To our knowledge, no DNA-based studies have been performed to estimate the genetic correlation between foot disorders and disease traits other than mastitis. Using genomic data from individual cows to estimate relationships may be more accurate than using pedigree data (Goddard, 2009; Hayes et al., 2009) because using genomic data reduces the standard error of the genetic correlation estimate (Visscher et al., 2014). Therefore, the aim of this study was to identify loci associated with susceptibility to multiple foot disorders and other common diseases, which could be coordinately used to inform breeding programs.

## Methods

All procedures were conducted in accordance with ethical standards set by the University of California, Davis and approved by the Institutional Animal Care and Use Committee (protocol #22099).

### Phenotypes

Five large commercial dairies (Dairies A–E, each with >1,000 cows) in Northern and Central California participated in this study. Phenotypes were derived from hoof trimming and other health records provided by the dairies beginning from the cows’ first lactation. Three hoof trimmers recorded the foot lesions used for phenotyping foot lesions: one who serviced Dairies A, B, and C; one who serviced Dairy D; and another who serviced Dairy E. Hoof trimmer experience and hoof trimming regimens were described previously (Lai et al., 2020, Lai et al., 2021). Foot disorders recorded included DD, foot rot, sole hemorrhage, SU, WLD, wall abscess, sole abscess, heel abscess, and laminitis. Other health events were also recorded by dairy personnel, which included diarrhea, displaced abomasum, ketosis, mastitis, metritis, milk fever, pneumonia, and retained placenta. For each foot or other health disorder, cases were defined as cows with at least one record of the disorder and controls were defined as cows that did not have records of the given foot or health disorder. Consequently, for each trait, controls included cows with disorders other than the disorder the cases had.

### Genotypes

Whole blood samples were obtained and the buffy coat was used to extract genomic DNA using the QIAGEN QIAamp DNA Blood Mini Kit (QIAGEN Inc., Valencia, CA). DNA samples were quantified using the NanoDrop (ND-2000 v3.2.1) spectrophotometer (Thermo Scientific, Wilmington, DE) and sent to GeneSeek (Lincoln, NE) for SNP genotyping on the high-density BovineHD BeadChip (777K SNPs, Illumina Inc., San Diego, CA). Genotype calls were made using Illumina’s GenCall algorithm. SNP genotypes from a subset of the cows used in this study were used in our past studies (Lai et al., 2020, Lai et al., 2021) and are publicly available at the NCBI Gene Expression Omnibus database (GEO series record GSE159157 and GSE165945), along with the additional samples from this study (GSE to be added when received from GEO). SNP genotypes were updated to the ARS-UCD1.2 assembly positions (Rosen et al., 2020) and quality-filtered in PLINK 1.9 (Purcell and Chang, 2015) by removing from further analyses SNPs and cows with <95% genotyping rate, SNPs with significant deviation from Hardy–Weinberg equilibrium (*p* < 1E-6) to exclude systematic genotyping errors, and SNPs with minor allele frequency <5% to exclude rare variants. Missing genotypes for each cow were imputed using BEAGLE 5.1 (Browning et al., 2018) using the other cows in the sample population as the reference population, an effective sample size of 58 for the United States Holstein cattle population (Makanjuola et al., 2020), and default parameters. Genetic similarity among cows was visualized in a multidimensional scaling (MDS) plot depicting the first two dimensions.

### Estimation of Genetic Correlation

Genetic correlation was estimated between each foot lesion and other health traits, including other foot lesions (e.g., genetic correlation was estimated between SU and WLD, SU and DD, SU and mastitis, and SU and metritis) using cows that had phenotypes for both traits and at least 40 case cows for each disease. PLINK 2.0 was used to filter cows by requiring phenotypes for both traits (Chang et al., 2015; Purcell and Chang, 2021). GCTA was used to calculate the genetic relatedness matrix (GRM), which was used with farm as a fixed effect to estimate the additive genetic variance and covariance between the two traits using two-trait GREML (Yang et al., 2011; Lee et al., 2012). Specifically, the phenotype for trait 1 of the *k*th cow from the *i*th farm at the *j*th SNP was modeled as

$$y_{1_{ijk}}=\mu_{1}+F_{1_{i}}+S_{1_{j}}+a_{1_{ik}}+\varepsilon_{1_{ijk}}$$

where $\mu_{1}$ was an unknown constant common to all cows for trait 1, $F_{1_{i}}$ was contribution of *i*th farm to the risk of disease, $S_{1_{j}}$ was the contribution of the *j*th SNP genotype to risk of the disorder, and $a_{1_{ik}}$ were the additive genetic effects assumed to be drawn from the multivariate normal density N (0, $A\sigma_{a}^{2}$ ), where *A* was the GRM. $\varepsilon_{1_{ijk}}$ was the residual term for trait 1. Similarly, the phenotype for trait 2 of the *k*th cow from the *i*th farm at the *j*th SNP was modeled using the same components for trait 2 as

$$y_{2_{ijk}}=\mu_{2}+F_{2_{i}}+S_{2_{j}}+a_{2_{ik}}+\varepsilon_{2_{ijk}}$$

The covariance of additive genetic effects was computed as a function of the numerator relationship matrix, and genetic correlation was calculated as the covariance of additive genetic effects divided by the product of the standard deviations of the genetic effect of traits 1 and 2. All genetic correlation estimates were transformed from the observed scale (0/1) to the underlying liability scale to account for case ascertainment using the prevalence of each disorder obtained from the literature (Oberbauer et al., 2013; USDA, 2018). Genetic correlation estimates were considered significantly different from zero if the estimate had *p* < 0.05 from the likelihood ratio test, and suggestive genetic correlation estimates were those with *p* < 0.1.

### Two-Trait Genome-Wide Association Study

Pairs of traits that had significant or suggestive genetic correlation estimates using the frequentist approach were evaluated further in two-trait GWAS to identify regions potentially contributing to both traits. Multi-trait association testing can improve the power to detect associations while accounting for population stratification (Banerjee et al., 2008; Korte et al., 2012; Zhou and Stephens, 2012, Zhou and Stephens, 2014) because the additional information from the covariance of traits is still informative, even if only one of the traits is associated with the genotype (Stephens, 2013). Two-trait genome-wide association analysis was performed to test for association of each SNP with at least one of the traits. A standardized GRM was constructed and included in the linear mixed model to account for relatedness and population stratification, and farm was included as a fixed effect to adjust for differences among farms. The linear mixed model association testing was conducted using the multivariate association testing function in GEMMA (Zhou and Stephens, 2012, Zhou and Stephens, 2014) using the same models for estimating genetic correlation. Bonferroni correction for multiple testing assumes that each test for SNP association with phenotype(s) is independent. However, because SNPs are not independent due to linkage disequilibrium (LD) between SNPs, the Genetic Type I error calculator (GEC) was used to calculate the effective number of markers after accounting for linkage disequilibrium between SNPs for use as the denominator in Bonferroni-corrected thresholds of significance (Li et al., 2012). Genome-wide significant SNPs were thus defined as those with likelihood ratio test (LRT) *p* < 0.05/M_e_ and suggestive SNPs, as those with LRT *p* < 1/M_e_ (Lander and Kruglyak, 1995). Manhattan and quantile–quantile plots were generated using the qqman package in R (R Development Core Team, 2010; Turner, 2014).

Because SNPs may not be causal for the traits but rather in LD with causal variants due in part to the long range linkage disequilibrium in cattle (Cai et al., 2019), SNPs were used to define LD blocks that were then mined for overlap with genes and previously defined QTL. SNPs in LD with significant and suggestive SNPs were used to define the start and end of LD blocks using a method similar to Richardson et al. (2016) and Twomey et al. (2019). SNPs that were within 5 Mb (upstream or downstream) and in LD (*R* ^2^ > 0.5) with significant or suggestive SNPs were considered belonging to the same LD block. LD blocks were queried in the region search of FAANGMine (FAANG, 2019) to identify genes within or overlapping with the LD block. LD blocks were also queried for overlap with previously defined QTL and associations related to feet and legs conformation traits and disease traits in the Cattle QTLdb (Hu et al., 2019) (version 46, accessed 4/30/2021).

## Results

### Descriptive Data

Hoof trimming records were available for 21,044 cows across the five dairies (distribution of records for each type of foot lesion is described in detail by Lai et al. (2021), of which 417 cows were selected for SNP genotyping as controls or cases for a certain foot lesion(s). Traits that were recorded at multiple dairies were used for genetic correlation estimation and two-trait GWAS, and the distribution of case/control phenotypes for each trait is listed in Table 1. All five dairies recorded SU, WLD, and DD foot disorders. All dairies except Dairy C also had health records available for phenotyping other health traits. These four dairies (Dairies A, B, D, and E) recorded mastitis, metritis, and pneumonia. Dairies A, B, and E also recorded ketosis, retained placenta, diarrhea, milk fever, and displaced abomasum. After excluding traits that have ≤40 cases, genetic correlation was estimated between each pair of foot disorders (SU, WLD, and DD) as well as each foot disorder with another health disorder (mastitis, metritis, retained placenta, milk fever, and pneumonia).

**TABLE 1:** Count of genotyped cows after quality filtering, split by cases for each foot disorder or other health condition and controls across the five dairies.

	Farm					Total
	A	B	C	D	E	
*Datasets for foot disorders*						
Sole ulcer						
Cases	44	8	4	71	25	152
Controls	138	70	26	23	0	257
White line disease						
Cases	48	13	7	33	16	117
Controls	134	65	23	61	9	292
Digital dermatitis						
Cases	19	22	30	30	5	106
Controls	163	56	0	64	20	303
*Datasets for other disorders*						
Mastitis						
Cases	89	66	NR	77	17	249
Controls	93	12	NR	17	8	130
Metritis						
Cases	57	51	NR	8	15	131
Controls	125	27	NR	86	10	248
Ketosis						
Cases	13	17	NR	NR	0	30
Controls	169	61	NR	NR	25	255
Retained placenta						
Cases	16	35	NR	NR	0	234
Controls	166	43	NR	NR	25	51
Diarrhea						
Cases	19	0	NR	NR	1	20
Controls	163	78	NR	NR	24	265
Milk fever						
Cases	61	9	NR	NR	0	70
Controls	121	69	NR	NR	25	215
Displaced abomasum						
Cases	1	17	NR	NR	2	20
Controls	181	61	NR	NR	23	265
Pneumonia						
Cases	2	4	NR	22	13	41
Controls	180	74	NR	72	12	338

NR, No records available; cows were excluded from analyses.

**FIGURE 1:**
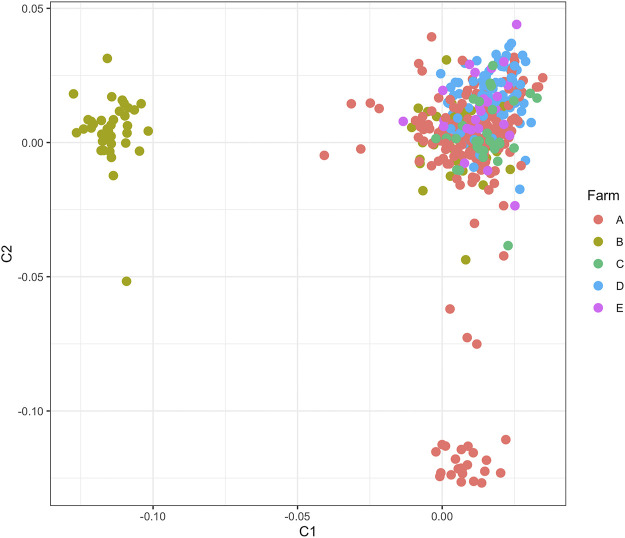
Multidimensional scaling plot showing the first two dimensions for 409 cows from the five dairies used in the estimation of genetic correlation and genome-wide association analyses.

Quality filtering removed eight cows and 218,306 SNPs, leaving 409 cows with 559,656 SNPs for analyses with case/control phenotypes presented in Table 1. The MDS plot indicated slight population stratification with a prominent center cluster, though cows were not strongly stratified by farm (Figure 1).

### Genetic Correlation Estimates

Of the pairs of traits for which genetic correlation was estimated, genetic correlation estimates between SU and WLD and between SU and DD were significantly different from zero (*p* < 0.05), and estimates between DD and mastitis, DD and milk fever, and SU and metritis were suggestive (*p* < 0.1, Table 2). Consequently, each pair of these traits was analyzed in two-trait GWAS.

**TABLE 2:** Genetic correlation estimates (and standard error, SE) between sole ulcer (SU), white line disease (WLD), digital dermatitis (DD), and other foot or health traits that were significantly or suggestively different from zero.

Trait 1	Trait 2	Genetic correlation (SE)	*p*	Significance
SU	DD	0.46 (0.25)	4.81E-02	^a^
SU	WLD	0.92 (0.46)	2.54E-02	^a^
DD	Mastitis	0.49 (0.36)	7.77E-02	^b^
DD	Milk fever	0.49 (0.39)	9.46E-02	^b^
SU	Metritis	0.7 (0.46)	5.22E-02	^b^

a Significant.

b Suggestive significance.

### Two-Trait Genome-Wide Association Analysis

**FIGURE 2:**
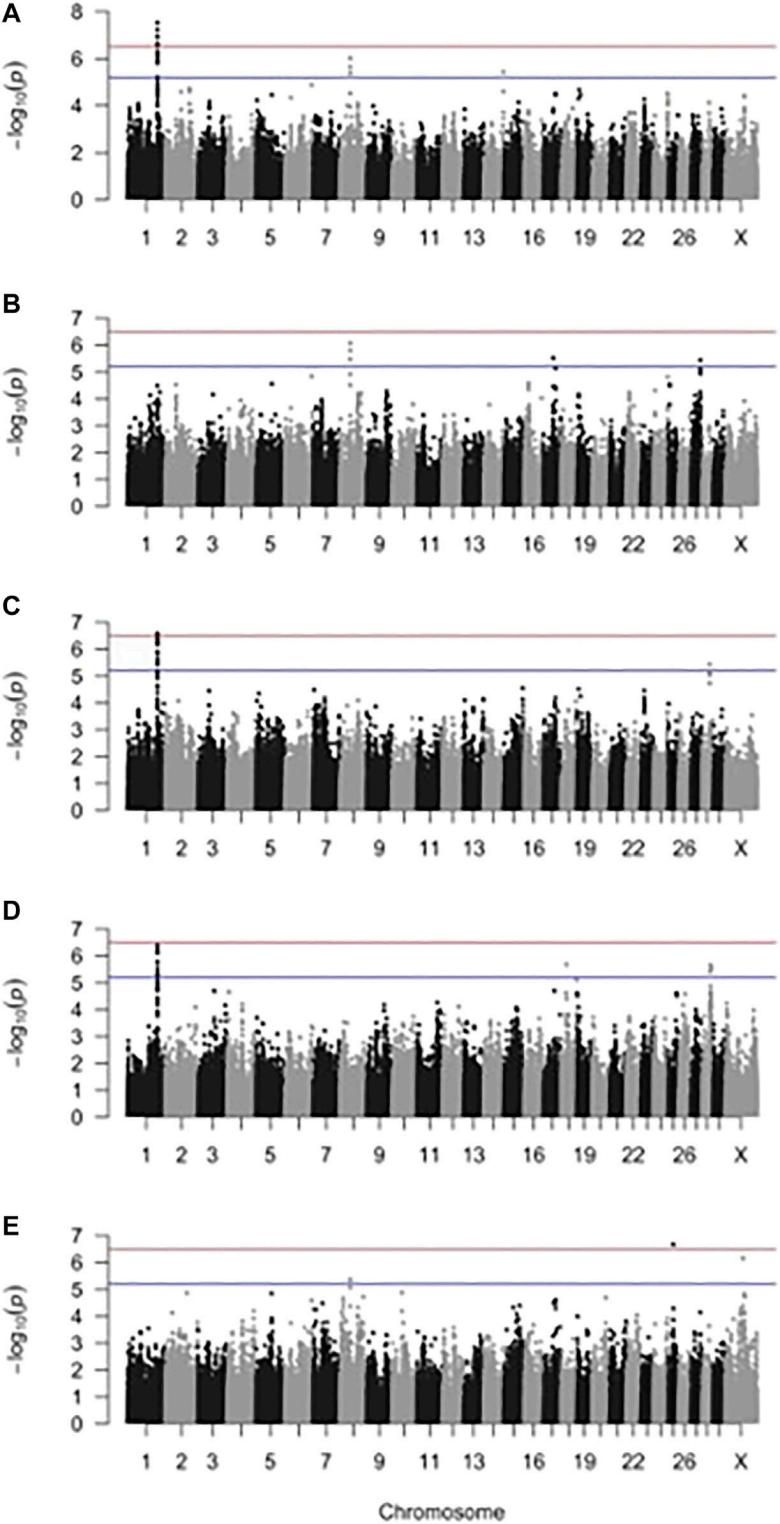
Manhattan plot for two-trait genome-wide association analysis of **(A)** sole ulcer (SU) and digital dermatitis (DD), **(B)** SU and white line disease (WLD), **(C)** DD and mastitis, **(D)** DD and milk fever, and **(E)** SU and metritis. The blue line indicates genome-wide suggestive significance, and the red line indicates genome-wide significance.

The effective number of markers after accounting for LD was 162,435 SNPs, corresponding to a suggestive threshold of 6.2 × 10^−6^ [5.2 on the −log_10_(*p*) scale] for genome-wide suggestive significance and 3.1 × 10^−7^ [6.5 on the −log_10_(*p*) scale] for genome-wide significance. Manhattan plots from the two-trait GWAS are shown in Figure 2. Genomic inflation factors ranged from 1.02 to 1.06 and, combined with the qqplots (Supplementary Figure S1), indicated that population stratification had been sufficiently accounted for (Price et al., 2010).

Significant and suggestive SNPs and the LD blocks they defined are shown in Table 3. Supplemental materials report the genes and QTL LD blocks (Supplementary Tables S1–S3). The GWAS that included DD as one of the traits (DD and mastitis, SU and DD, and DD and milk fever) identified significant and suggestive SNPs belonging to the same LD block at BTA1:125550933–125822143. For the DD and mastitis and DD and milk fever GWAS, the peak on BTA1 reached or approached genome-wide significance despite the genetic correlation estimate only reaching suggestive significance (Table 1 and Supplementary Table S1). GWAS that included SU as one of the traits (that is, between SU and WLD, SU and DD, and SU and metritis) all identified suggestive SNPs in an LD block at BTA8:42926603–44642925. Other SNP associations were unique to the pair of traits for which the GWAS was performed such that SNPs that were associated in a certain GWAS for a pair of traits were not associated in other GWAS for other pairs of traits. For instance, the LD block on BTA14 detected from the GWAS for SU and DD was only detected in the SU and DD GWAS and not detected in any other of the comparisons such as that for SU and WLD, DD and mastitis, DD and milk fever, and SU and metritis. Although SU and WLD were strongly genetically correlated (0.92), the suggestive SNPs identified in the two-trait GWAS had opposite effect signs between the two traits: if the effect of the SNP was positive for SU, it was negative for WLD and *vice versa* (Table 3 and Supplementary Table S1). The LD blocks defined from all the two-trait GWAS overlapped with 83 protein-coding genes, some functionally relevant to the etiology of the disorders (Supplementary Table S2).

**TABLE 3:** Linkage disequilibrium (LD) blocks and the most significant SNP within the LD block defined from the two-trait genome-wide association analyses of sole ulcer (SU), white line disease (WLD), and digital dermatitis (DD) paired with each other and other health disorders.

Dataset (trait 1 and trait 2)	BTA	LD block start (bp)	LD block end (bp)	LD block length (kb)	Most significant SNP	SNP position (bp)	Minor/Major allele	Effect size for trait 1	Effect size for trait 2	Variance matrix for beta effects			*p*	
										Variance of effect size for trait 1	Covariance between effect sizes of trait 1 and 2	Variance of effect size for trait 2		
SU and DD	1	125550933	125822143	271.21	BovineHD0100035768	125563251	A/G	1.85E-01	4.64E-03	1.09E-03	3.13E-05	1.46E-03	2.58E-07^a^
	8	42926603	44642925	1716.322	BovineHD0800013406	44628587	T/C	2.05E-01	-8.65E-02	2.30E-03	6.91E-04	2.99E-03	3.06E-06^b^
	14	81655298	81664096	8.798	BovineHD1400023802	81655298	G/T	-3.00E-02	1.58E-01	1.05E-03	3.17E-04	1.21E-03	3.68E-06^b^
SU and WLD	8	42926603	44642925	1716.322	BovineHD0800013408	44632844	G/T	4.90E-02	1.91E-01	9.97E-04	-9.75E-06	1.14E-03	2.11E-07^a^
	17	41328134	41328134	0	BovineHD1700011766	41328134	C/T	-1.21E-01	1.32E-01	1.12E-03	8.92E-05	1.23E-03	7.07E-07^b^
	27	37518206	38922466	1,404.26	BovineHD2700011209	38898651	T/C	-1.21E-01	1.32E-01	1.12E-03	8.92E-05	1.23E-03	7.07E-07^b^
	27	37518206	38922466	1,404.26	BovineHD2700011210	38901656	G/A	-1.21E-01	1.32E-01	1.12E-03	8.92E-05	1.23E-03	7.07E-07^b^
DD and mastitis	1	125550933	125822143	271.21	BovineHD0100035835	125691064	A/G	1.02E-01	1.72E-01	1.06E-03	1.61E-04	9.47E-04	2.98E-08^a^
	28	33357088	33385923	28.835	BovineHD2800009006	33385923	C/T	9.39E-02	1.62E-01	1.01E-03	1.59E-04	9.07E-04	1.20E-07^a^
DD and milk fever	1	125550933	125822143	271.21	BovineHD0100035785	125585828	C/T	8.88E-02	1.64E-01	1.01E-03	1.61E-04	9.08E-04	1.19E-07^a^
	1	125550933	125822143	271.21	BovineHD0100035800	125624770	A/C	8.88E-02	1.64E-01	1.01E-03	1.61E-04	9.08E-04	1.19E-07^a^
	18	24087895	24329676	241.781	BovineHD1800007458	24087895	C/T	6.46E-02	1.50E-01	9.06E-04	1.51E-04	8.12E-04	8.13E-07^b^
	28	34935232	35093950	158.718	BTB-00987935	35093950	G/T	6.46E-02	1.50E-01	9.06E-04	1.51E-04	8.12E-04	8.13E-07^b^
	28	35837718	36740498	902.78	BovineHD2800010153	36916301	C/T	6.52E-02	1.49E-01	8.93E-04	1.47E-04	8.00E-04	7.86E-07^b^
	28	35837718	36740498	902.78	BovineHD2800010156	36926419	C/T	6.52E-02	1.49E-01	8.93E-04	1.47E-04	8.00E-04	7.86E-07^b^
	28	38776483	42482917	3706.434	BovineHD2800011177	40061500	G/A	8.15E-02	1.45E-01	9.42E-04	1.53E-04	8.54E-04	1.40E-06^b^
SU and metritis	8	42926603	44642925	1716.322	BovineHD0800013412	44642925	A/C	6.16E-02	1.57E-01	9.06E-04	1.50E-04	8.03E-04	2.22E-07^a^
	25	22127459	22966511	839.052	BovineHD2500006264	22127459	A/G	1.86E-01	-2.77E-02	1.52E-03	2.99E-04	1.49E-03	4.09E-06^b^
	X	75319558	75610976	291.418	BovineHD3000022324	75393744	A/G	2.01E-01	-1.21E-02	1.49E-03	2.90E-04	1.46E-03	9.61E-07^b^

a Genome-wide significance.

b Genome-wide suggestive significance.

## Discussion

We estimated the genetic correlation between common foot disorders (DD, SU, and WLD) and other health traits (mastitis, metritis, milk fever, retained placenta, and pneumonia). For pairs of traits having significant or suggestive genetic correlation, the loci that were contributing to the correlation were examined using two-trait GWAS. To our knowledge, this is the first study to estimate genetic correlation between foot disorders and diseases other than mastitis from individual-level genotype data rather than pedigree data and identify loci potentially contributing to the correlation. Genetic correlation estimates that were significant or suggestive included SU or DD as one of the traits and estimates were positive, indicating a favorable genetic correlation between pairs of disease traits such that genetic selection against one disease will lead to selection against the other disease. Significant and suggestive SNPs were detected in the same regions on BTA1 and BTA8 for two-trait GWAS datasets that had DD and SU as one of the traits, respectively, suggesting that DD and SU were driving the association in these genomic regions. Other significant and suggestive SNPs were specific to the dataset from which they were detected and not detected in GWAS for other pairs of traits.

Compared to previous estimates of genetic correlation between foot disorders and other health traits, estimates from this study were higher and had larger standard errors. Previous estimates of genetic correlation between foot disorders and mastitis or somatic cell count were significantly different from zero (0.15–0.35) (Koenig et al., 2005; Buch et al., 2011) or close to zero (Gernand et al., 2012), whereas we estimated the genetic correlation between DD and mastitis at 0.49 (SE = 0.36). The genetic correlation between SU and WLD was 0.92 (SE = 0.46) and substantially higher than previous estimates, which ranged from 0.41 to 0.60 (van der Linde et al., 2010). The estimates of genetic correlation from this study were higher likely because controls were shared between the two traits and the proportion of cows with DD and/or SU was higher than for other disorders. Because case cows were sampled primarily for DD and SU and other disorders were phenotyped after sampling DD and SU cases, cases for other disorders frequently also had DD and/or SU. This overrepresentation of cases with DD and/or SU in addition to the disorder of interest likely inflated genetic correlation estimates, which the correction for case ascertainment was unable to overcome. The strong genetic correlation between SU and WLD in this study implied that whichever other traits SU is correlated with, WLD will also be correlated with and *vice versa*; however, SU was correlated with metritis and DD whereas WLD was not correlated with either disorder. This divergence would suggest that although SU and WLD share a genetic component, differences exist in the location or direction of the effect for susceptibility loci between SU and WLD, as indicated by the opposite signs of suggestive SNP effects between the traits and the lack of association of WLD to metritis or DD in the two-trait GWAS.

Compared to our previous one-trait GWAS for DD and SU (Lai et al., 2020, 2021), the two-trait GWAS detected the same LD block on BTA1 for DD and a different LD block on BTA8 for SU. Specifically, the LD block at BTA1:125550933–125822143 common to all datasets that had DD as one of the traits (DD and mastitis, SU and DD, and DD and milk fever) was the same LD block detected in our previous single-trait DD GWAS (Lai et al., 2020). The increase in significance of association also suggests that this region may play a role in both infectious (mastitis) and metabolic (SU and milk fever) disorders. Infectious and metabolic disorders have been observed to coincide and happen most frequently during the early lactation period (USDA, 2018), potentially due to a common cause. Some have attributed the cause of higher incidence of infectious and noninfectious foot disorders during early lactation to the extreme negative energy balance during this period (Collard et al., 2000; Gernand et al., 2013). Accordingly, it is thought that cows that are better able to cope with the energy requirements during this period are consequently less susceptible to metabolic and infectious disorders, a hypothesis supported by the association of a more robust adaptive immune response with lower incidence of metabolic disease during the periparturient period (Thompson-Crispi et al., 2012). Another common LD block at BTA8:42926603–44642925 was detected from the two-trait GWAS with SU as one of the traits (SU and WLD, SU and DD, and SU and metritis). This LD block was 30 Mb upstream of the LD block on BTA8 observed in our previous one-trait SU GWAS (Lai et al., 2021). Our previous GWAS used the same SU cases but only sound, older (>6.0 years old) cows as controls, whereas the present GWAS included controls with foot disorders other than the foot disorder the cases had. Consequently, the present GWAS controlled for other foot disorders that the cases had such that associated regions were more likely for SU specifically and not for other foot disorders correlated with SU, whereas the single-trait GWAS used the most phenotypically divergent cows as controls to maximize the power to detect genetic differences.

In addition to detecting the same regions as the one-trait GWAS, the two-trait GWAS detected other regions that were not detected in the one-trait GWAS for DD, SU, and/or WLD. The DD and mastitis GWAS detected a region on BTA28 that the DD GWAS did not find (Lai et al., 2020). The SU and DD two-trait GWAS found a region on BTA14 that was not identified in either the DD or SU one-trait GWAS (Lai et al., 2020, Lai et al., 2021). The suggestive SNP on BTA17 from the two-trait GWAS of SU and WLD was not in LD with other SNPs and not detected in the one-trait GWAS for SU or WLD (Lai et al., 2021). The two-trait GWAS for DD and milk fever detected regions on BTA18 and 28 that were not detected in the one-trait DD GWAS (Lai et al., 2020). Finally, the two-trait GWAS for DD and milk fever detected regions on BTA18 and 28 that were not detected in the one-trait DD GWAS (Lai et al., 2020). The genetic correlation between the two traits may have provided additional power to detect these associations that were underpowered in the one-trait GWAS.

The LD blocks defined from each dataset overlapped with genes and/or QTL that were functionally relevant to both traits. Genes having functions that were considered relevant to the etiology of each disorder were defined for each trait (Supplementary Table S4) and included those with a role in immune function, hair follicle morphology, hair density, skin integrity, fibroblast proliferation, bone growth and mineralization, adipose and body fat, and glucose metabolism. The LD block at BTA1:125550933–125822143 from the GWAS that included DD as one of the traits contained *SLC9A9* (solute carrier family 9 member A9) (Lai et al., 2020), which has been implicated in multiple sclerosis in humans through its role in regulating T-cell activation and differentiation to a induce a proinflammatory response (Esposito et al., 2015). Notably, the DD and mastitis LD block at 1:125839933–125852054 overlapped with a QTL associated with length of productive life (Cole et al., 2011), corroborating the shorter productive life associated with DD and mastitis susceptibility (Shabalina et al., 2020). Previous estimates of genetic correlation between foot lesion traits and productive life were close to zero (Dhakal et al., 2015), suggesting that uncorrelated traits may still share pleiotropic loci, as observed previously between various production, fertility, and conformation traits (Xiang et al., 2017). This LD block on BTA1 from the DD and mastitis GWAS and the LD block on BTA27 from the SU and WLD GWAS both overlapped with QTL for feet and legs conformation traits (Cole et al., 2011), and could be a pleiotropic locus contributing to the genetic correlation between feet and legs conformation and susceptibility to foot lesions (Häggman and Juga, 2013; Malchiodi et al., 2017; Ring et al., 2018), though this genetic correlation is too low to justify indirect selection on lameness using feet and legs conformation traits (Van Raden et al., 2021). The LD blocks from the GWAS for SU and DD, SU and WLD, and SU and metritis overlap with QTL for infectious disease traits (tuberculosis susceptibility, clinical mastitis, and somatic cell score/count) and blood cortisol, which may reflect the interplay of the stress from the negative energy balance during the periparturient period possibly potentiating metabolic and infectious foot disorders (Supplementary Table S3). Cows with SU tend to exhibit markers of chronic inflammation compared to cows without SU (O’Driscoll et al., 2015), though it is unclear if SU causes inflammation, *vice versa*, or both are the product of stress.

The main limitations of this study were the small sample size of genotyped cows and the variation in the number of case cows across the various disorders. At the expense of a larger sample size, we minimized the environmental variation by constraining the sample population to cows to a small geographical region under similar management and nutrition practices and minimized the number of hoof trimmers to reduce variation in phenotyping foot lesions. Minimizing environmental and consistent phenotyping improves the power to detect significant genetic correlation; however, the resulting small sample size limited the accuracy of genetic correlation estimates. For instance, one workaround for the inflation of genetic correlation estimates due to shared controls is to randomly partition the controls between the two traits before estimating genetic correlation; however, the small sample size prevented using this approach. The small sample size also limited the benefit of using genomic data instead of pedigree data to estimate genetic correlation. Although using genomic data to estimate relationships may be more accurate than using pedigree data (Goddard, 2009; Hayes et al., 2009) due to reduced standard error of the genetic correlation estimate (Visscher et al., 2014), the standard error of the genetic correlation estimates in this study was large, reflecting the limited sample size. The reduction in standard error from using genomic data would be more appreciable in larger sample sizes. Ascertainment bias for cows with DD and SU but not the other disorders likely led to an overrepresentation of cows with DD and/or SU in the dataset, resulting in inflated estimates between DD or SU and the other disorders. Despite the inflated and large standard errors of the genetic correlation estimates, some estimates were significantly or suggestively different from zero and provided grounds for further investigation of SNPs contributing to the correlation using the two-trait GWAS. The sample size also provided sufficient power in the two-trait GWAS to detect significant and suggestive SNPs that defined LD blocks overlapping with functionally relevant genes and QTL, similar to previous GWAS using similar small sample sizes (∼400 cows) and high-density SNP genotypes (Buzanskas et al., 2017; Lehner et al., 2018).

## Conclusion

A genomic relatedness matrix calculated from SNP genotypes was used to estimate genetic correlation between individual foot disorders (DD, SU, and WLD) and other health disorders (mastitis, metritis, milk fever, retained placenta, and pneumonia). The positive estimates of genetic correlation between individual foot disorders and other health disorders indicate that direct selection against foot disorders will not increase the incidence of other health disorders and may in fact reduce their prevalence. Genomic assessment for pairs of traits that were genetically correlated revealed multiple associated regions. Whereas some of these chromosomal regions were shared across multiple pairs of traits that included SU or DD as one of the traits, others were unique to the pair of traits, indicating the complexity of genetic contributions within and between traits. The LD blocks defined from associated SNPs included protein-coding genes and QTL that were functionally relevant to both traits, suggesting that selection for markers in these LD blocks would affect susceptibility to both traits.

## Data Availability

The datasets presented in this study can be found in online repositories. The names of the repository/repositories and accession number(s) can be found at: https://www.ncbi.nlm.nih.gov/geo/, GSE159157, GSE165945, and GSE186266.

## Abbreviations

*Bos taurus* autosome (BTA), digital dermatitis (DD), genetic relatedness matrix (GRM), Genetic Type I error calculator (GEC), genome-wide association study (GWAS), likelihood ratio test (LRT), linkage disequilibrium (LD), minor allele frequency (MAF), quantitative trait loci (QTL), single nucleotide polymorphism (SNP), sole ulcers (SU), white line disease (WLD).
