# Supplementary material for: Pleiotropic Loci Associated With Foot Disorders and Common Periparturient Diseases in Holstein Cattle
Source: Front Genet. 2021 Dec 6;12:742934. doi: 10.3389/fgene.2021.742934 (PMC8685441; doi:10.3389/fgene.2021.742934)
Supplement: Supplementary file 1 [file DataSheet1.pdf]

Supplementary Material

Supplementary material for “Pleiotropic loci associated with foot disorders and common periparturient diseases in Holstein cattle” (Lai et al. 2021).

1 Supplementary Tables

Table S1. Significant and suggestive SNPs detected in the two-trait linear mixed model association analysis and the linkage disequilibrium (LD) blocks they defined for sole ulcers (SU) and digital dermatitis (DD), SU and white line disease (WLD), DD and mastitis, DD and milk fever, and SU and metritis.

Table S1 is available in the Excel workbook containing all supplemental tables.

Table S2. Genes that overlapped with linkage disequilibrium blocks defined from the genome-wide association analyses for sole ulcers (SU) and digital dermatitis (DD), SU and white line disease (WLD), DD and mastitis, DD and milk fever, and SU and metritis. Phenotypes associated with mouse knockout models of protein-coding genes and the functional relevance of the phenotype to the dataset, if applicable, are also listed. (See Table S4 for which phenotypes were considered functionally relevant for each disorder.)

Table S2 is available in the Excel workbook containing all supplemental tables.

Table S3. Previously defined quantitative trait loci (QTL) or associations overlapping with or in the linkage disequilibrium (LD) blocks defined from the two-trait genome-wide association analyses for sole ulcers (SU) and digital dermatitis (DD), SU and white line disease (WLD), DD and mastitis, DD and milk fever, and SU and metritis that were functionally relevant to at least one of the two traits.

Table S3 is also available in the Excel workbook containing all supplemental tables.

	LD block location					
Dataset	BTA	Start (bp)	End (bp)	Length (kb)	Overlapping QTL or association	Reference
DD and mastitis	1	125550933	125822143	271.2	Digital dermatitis	(Lai et al., 2020)
	1	125839933	125852054	12.1	Feet and legs conformation	(Cole et al., 2011)
					Length of productive life	(Cole et al., 2011)
	28	33357088	33385923	28.8	No overlap with functionally relevant QTL/associations	
SU and WLD	8	42926603	44642925	1716.3	Blood cortisol level	(Chen et al., 2020)
					Bovine tuberculosis susceptibility	(Richardson et al., 2016; González-Ruiz et al., 2019)
	17	41328134	41328134	0	No overlapping QTL/associations	
	27	37518206	38922466	1404.3	Foot angle	(Cole et al., 2011)
					Length of productive life	(Cole et al., 2011)
					Net merit	(Cole et al., 2011)
					Rear leg placement - rear view	(Cole et al., 2011)
					Rear leg placement - side view	(Cole et al., 2011)
					Somatic cell score	(Cole et al., 2011)
SU and DD	1	125550933	125822143	271.2	Digital dermatitis	(Lai et al., 2020)
	8	42926603	44642925	1716.3	Blood cortisol level	(Chen et al., 2020)
					Bovine tuberculosis susceptibility	(Richardson et al., 2016; González-Ruiz et al., 2019)
	14	81655298	81664096	8.8	No overlapping QTL/associations	
DD and milk fever	1	125550933	125822143	271.2	Digital dermatitis	(Lai et al., 2020)
	18	24087895	24329676	241.8	No overlapping QTL/associations	
	28	34935232	35093950	158.7	No overlapping QTL/associations	
	28	35837718	36740498	902.8	No overlapping QTL/associations	
	28	38776483	42482917	3706.4	No overlapping QTL/associations	
SU and metritis	8	42926603	44642925	1716.3	Blood cortisol level	(Chen et al., 2020)
					Bovine tuberculosis susceptibility	(Richardson et al., 2016; González-Ruiz et al., 2019)
	25	22127459	22966511	839.1	Bovine respiratory disease susceptibility	(Neupane et al., 2018)
	X	75319558	75610976	291.4	No overlapping QTL/associations	

Table S4. Phenotypes that were considered functionally relevant digital dermatitis (DD), sole ulcers (SU), white line disease (WLD) and other health disorders.

	Infectious			Noninfectious		
**Gene function**	**DD**	**Mastitis**	**Metritis**	**SU**	**WLD**	**Milk fever**
Adipose/fat				x	x	
Bone				x	x	x
Cartilage				x	x	
Chondrocyte				x	x	
Immune	x	x	x			
Hair	x					
Skin	x					
Collagen				x	x	
Glucose metabolism				x	x	
Fibroblast proliferation				x	x	

2 Supplementary Figures

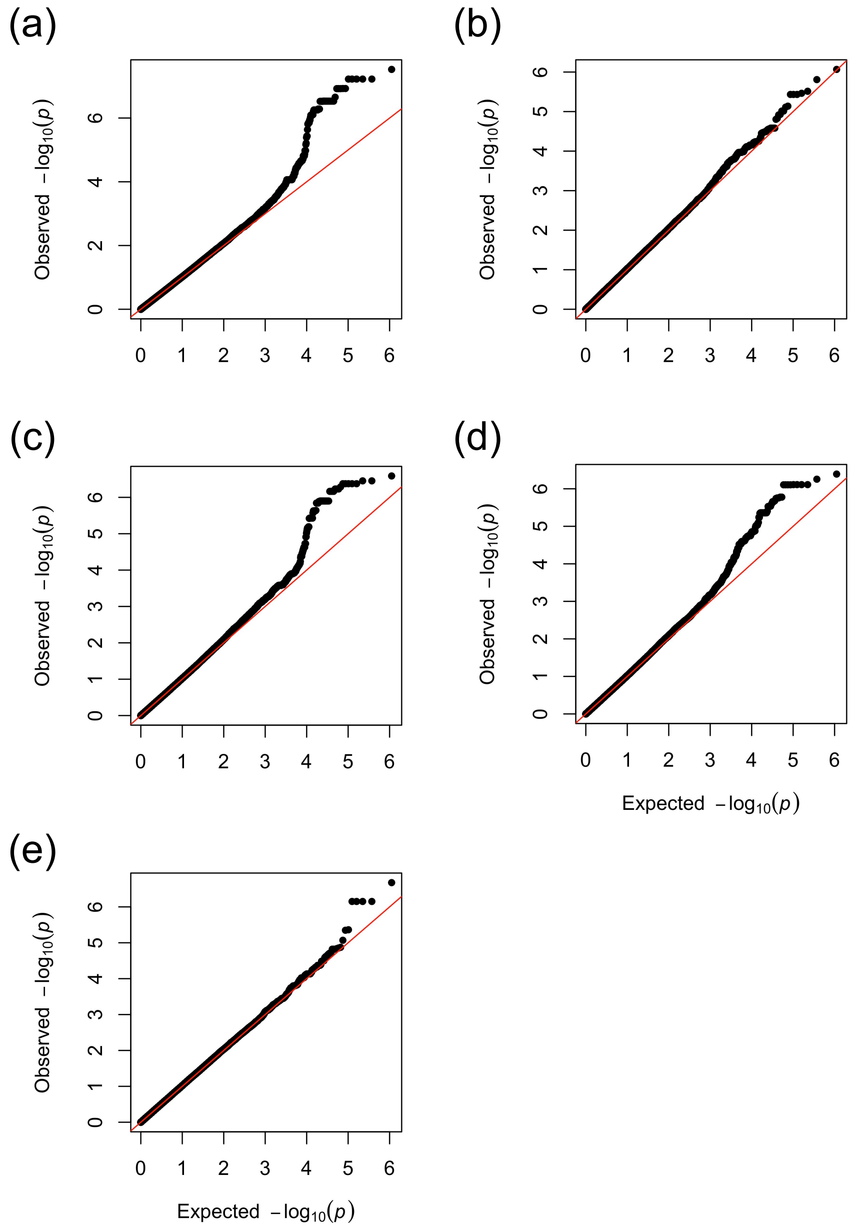

Figure S1. Quantile-quantile plot for two-trait genome-wide association analysis of (a) sole ulcer (SU) and digital dermatitis (DD), (b) SU and white line disease (WLD), (c) DD and mastitis, (d) DD and milk fever, and (e) SU and metritis.
